# Non-invasive risk-assessment and bleeding prophylaxis with IVIG in pregnant women with a history of fetal and neonatal alloimmune thrombocytopenia: management to minimize adverse events

**DOI:** 10.1007/s00404-020-05618-y

**Published:** 2020-06-04

**Authors:** Sandra Wienzek-Lischka, Angelika Sawazki, Harald Ehrhardt, Ulrich J. Sachs, Roland Axt-Fliedner, Gregor Bein

**Affiliations:** 1 Institute for Clinical Immunology and Transfusion Medicine, Justus-Liebig-University Giessen, Langhansstr. 7, 35392 Giessen, Germany; 2 Department of Obstectrics/Gynaecology, Justus-Liebig-University Giessen, 35392 Giessen, Germany; 3 Department of General Pediatrics and Neonatology, Justus-Liebig-University Giessen, 35392 Giessen, Germany; 4 German Center for feto-maternal Incompatibility, 35392 Giessen, Germany

**Keywords:** Fetal and neonatal alloimmune thrombocytopenia (FNAIT), Non-invasive bleeding prophylaxis, IVIG, Adverse events

## Abstract

**Introduction:**

In pregnant women with a history of fetal and neonatal alloimmune thrombocytopenia (FNAIT), prenatal intervention in subsequent pregnancies may be required to prevent fetal bleeding. Several invasive and non-invasive protocols have been published: amniocentesis for fetal genotyping, fetal blood sampling for the determination of fetal platelet count, intrauterine platelet transfusions, and weekly maternal i.v. immunoglobulin (IVIG) infusion with or without additional corticosteroid therapy. This is the first retrospective study that report the experience with a non-invasive protocol focused on side effects of maternal IVIG treatment and neonatal outcome.

**Methods:**

Pregnant women with proven FNAIT in history and an antigen positive fetus were treated with IVIG (1 g/kg/bw) every week. To identify potential IVIG-related hemolytic reactions isoagglutinin titer of each IVIG lot and maternal blood count were controlled. IVIG-related side effects were prospectively documented and evaluated. Furthermore, ultrasound examination of the fetus was performed before starting IVIG administration and continued regularly during treatment. Outcome of the index and subsequent pregnancy was compared. Corresponding data of the newborns were analyzed simultaneously.

**Results:**

IVIG was started at 20 weeks of gestation (median). Compared to the index pregnancy, platelet counts of the newborns were higher in all cases. No intracranial hemorrhage occurred (Index pregnancies: 1 case). Platelet counts were 187 × 10^9^/l (median, range 22–239, 95% CI) and one newborn had mild bleeding. No severe hemolytic reaction was observed and side effects were moderate.

**Conclusion:**

Among pregnant women with FNAIT history, the use of non-invasive fetal risk determination and maternal IVIG resulted in favorite outcome of all newborns. Invasive diagnostic or therapeutic procedures in women with a history of FNAIT should be abandoned.

## Introduction

Fetal and neonatal alloimmune thrombocytopenia (FNAIT) is caused by maternal alloantibodies that are directed against fetal human platelet antigens (HPAs) inherited from the father. After transplacental transport, these alloantibodies can induce mild to severe thrombocytopenia of the fetus or newborn. One of 1000 neonates is affected [1]. In most cases of Caucasian women, FNAIT is caused by antibodies against the HPA-1a antigen, followed by anti-HPA-5b (in more than 90% of NAIT cases). Occasionally, two HPA antibodies are present. Anti-HPA-15b antibodies are found in 3–4% of FNAIT cases [2]. Antibodies against HPA-4b occur predominantly in mothers of Asian descent [2]. Unlike RhD-incompatibility, FNAIT can occur during the first pregnancy [3]. One of the most devastating consequences is (fetal) intracranial hemorrhage (ICH) and/or death, which occur in 10% of symptomatic infants. A recent cohort study characterized pregnancies where the fetus or newborn was diagnosed with FNAIT and suffered from ICH [4]. The majority of bleedings (54%) occurred before 28 gestational weeks and often affected the first born child (63%). No cases of intrapartum ICH bleedings were confirmed and only two of 43 bleedings occurred after delivery.

Subsequent pregnancies should be managed in specialized centers with physicians experienced in FNAIT diagnosis and management to prevent ICH or other severe bleedings in the fetus or newborn. A step-wise procedure is recommended. In subsequent pregnancies it is required to determine whether the fetus is carrier of the antigen against which the maternal antibody is directed. The HPA genotype of the father should be first determined. If the father is homozygous all subsequent fetuses will inherit the implicated antigen and will be incompatible. In case of paternal heterozygosity the fetus will have a 50% chance for inheriting the implicated allele. In this case, prenatal genotyping is needed [2]. Fetal genotyping should be performed non-invasively using cell-free fetal DNA in maternal plasma. Invasive fetal diagnosis by chorionic villous sampling or amniocentesis have associated risks and may booster the maternal alloantibody [5, 6]. Recently, we developed a non-invasive diagnostic test using next-generation sequencing of maternal cell free plasma DNA for determination of fetal HPA-status [7]. When a fetus has been determined to be at risk, prophylactic intervention should be initiated. Historically, the management of subsequent pregnancies included serial fetal blood sampling (FBS) to determine the fetal platelet count and intrauterine transfusion of platelets.

FBS and intrauterine platelet transfusion are associated with severe adverse events like booster of alloimmunization, fetal bradycardia, bleeding complication in the fetus, intrauterine death or emergency Caesarean section [5, 6]. Furthermore, platelet transfusions are needed regularly, due to the short life time of transfused platelets, increasing the risk for fetal loss [8]. Non-invasive fetal and neonatal bleeding prophylaxis by weekly maternal i.v. Immunoglobulin infusion was introduced in 1988 by Bussel et al. [9]. Since then, several studies have been published. However, due to the rarity of severe FNAIT, these studies show considerable heterogeneity in management strategies applied including fetal surveillance with or without FBS, IVIG dosing, and IVIG with or without corticosteroids. A recent systematic review suggests that first-line antenatal management in FNAIT is weekly IVIG administration [5]. Most studies reported fetal outcome and did not systematically evaluate maternal adverse effects of weekly IVIG infusion.

In view of published results of a case series that did not report any therapeutic effect of maternal IVIG prophylaxis on fetal platelet counts [10] and lack of systematic evaluation of potential adverse effects of IVIG, we retrospectively evaluated the antenatal management of FNAIT in 12 subsequent patients that were treated according to a standardized local protocol. The results of this study demonstrate that a completely non-invasive management by weekly IVIG administration without corticosteroids is safe and effective. Adverse effects of IVIG are common but not severe.

## Materials and methods

### Patients

**Fig. 1:**
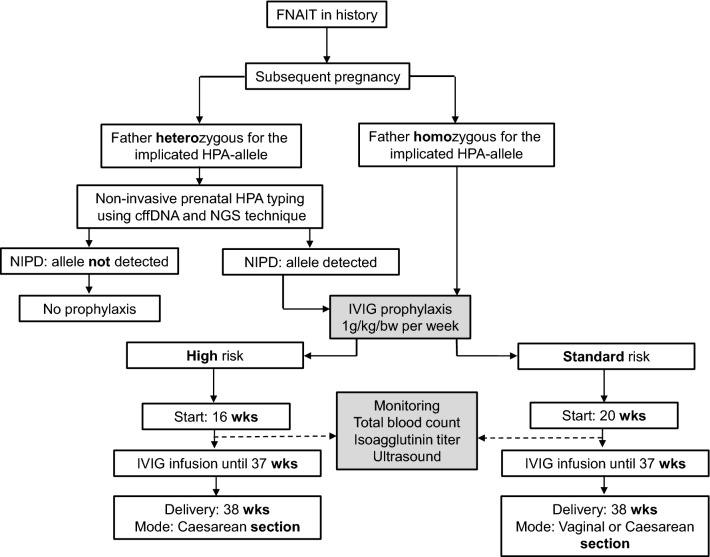
Prophylaxis scheme “Giessen Protocol”. *FNAIT* fetal and neonatal alloimmune thrombocytopenia, *HPA* human platelet antigen, *NIPD* non-invasive prenatal diagnostics, *IVIG* intravenous immunoglobulin, *bw* body weight, *wks* weeks of gestation

13 Pregnant women with proven FNAIT in history who were treated from August 2013 to April 2017 with i.v. Immunoglobulin (IVIG) prophylaxis (protocol described in textbox 1 and visualized in Fig. 1) were enrolled in this retrospective study. Inclusion criteria were a history of FNAIT in previous pregnancy, detection of anti-HPA alloantibodies before start of IVIG prophylaxis, and an antigen positive fetus. High risk patients had intracranial hemorrhage (ICH) or fetal death in previous pregnancies. In the group with standard risk no ICH or fetal death occurred. In case of paternal heterozygosity for the implicated HPA-antigen, non-invasive fetal genotyping using next-generation sequencing [7] was performed to determine whether the fetus was at risk (results not shown).
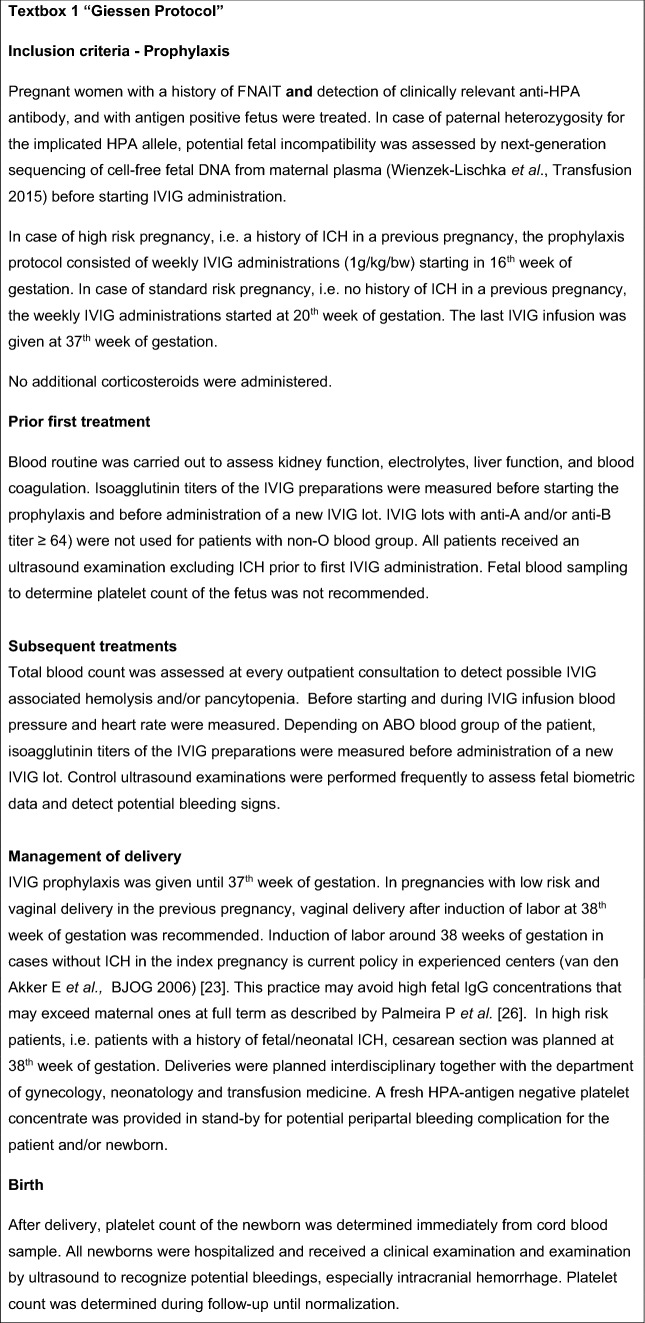
.

We excluded one woman with a history of fetal death due to intracranial hemorrhage who asked to do so, in addition to IVIG prophylaxis, for invasive diagnosis by serial fetal blood sampling and intrauterine platelet transfusions.

### Data sources

Patient’s records were evaluated retrospectively. Demographic characteristics, previous pregnancies, start and number of treatments, laboratory reports and levels of isoagglutinin titer of the administered IVIG preparations, maternal side effects of IVIG administration, results of all ultrasound examinations to assess fetal biometric data and to detect potential bleeding signs were extracted. Beside antenatal baseline characteristics, postnatal data were collected and included mode of delivery, gestational age at birth, birth weight, sex, APGAR score, platelet count at birth (cord blood), presence of bleeding signs, and platelet transfusions.

### Isoagglutinin antibody titer assessment

To minimize IVIG-related adverse events, especially IVIG-related hemolysis/pancytopenia, isoagglutinin titer of each lot that was intended to be given to a patient with blood group A, B or AB was assessed. Serially diluted IVIG was tested against autologous red blood cells in an anti-human globulin gel column test (Biorad, Munich, Germany). Lots with isoagglutinin titers ≥ 64 were not used.

### Statistical analysis

Data were reported as mean or median values, with minimum, maximum, and 95% confidence interval (CI) or numerical values. The Mann–Whitney *U* test was used for comparison of neonatal platelet counts of preceding pregnancy and pregnancy with IVIG prophylaxis. Two-way Anova was used for comparison of hemoglobin levels during IVIG prophylaxis in pregnant women with blood group O versus blood group non-O. Analyses were performed with Prism8, GraphPad Software, San Diego, CA, USA.

### Study approval

The Ethical Committee of the Medical Faculty of the Justus-Liebig-University, Giessen, Germany, approved this study on 21th April 2017 (Votum No. 63/17).

## Results

### Maternal outcome

**Fig. 2:**
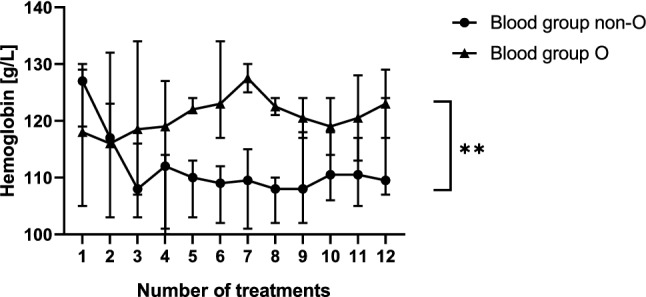
Comparison of hemoglobin levels in pregnant women with blood group non-O (*n* = 7) versus pregnant women with blood group O (*n* = 5) during prophylaxis with intravenous immunoglobulin (IVIG). Median, IQR, **p value 0.0013, *p* value is based on two-way Anova test

**Fig. 3:**
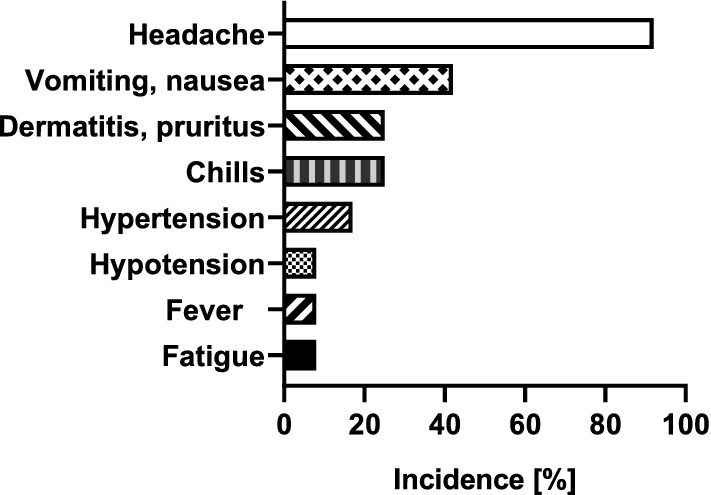
Incidence of adverse events during prophylaxis with i.v. immunoglobulin (*n* = 12 patients)

11 out of 12 patients were immunized against HPA-1a antigen, one patient against HPA-15a antigen. IVIG prophylaxis was started at 20 weeks of gestation (median, range 20–31) and continued until 37th or 38th week of gestation. 15 weekly IVIG infusion episodes of 1 g/kg/bw were given (median, range 6–19, Table 1). In one patient with high body mass index (44 kg/m^2^), the dose was split to two weekly doses of 0.5 g/kg/bw due to side effects (nausea, vomiting). Six patients had blood group A, one blood group B and five blood group O. Anti-A and anti-B titers of IVIG preparations varied from lot to lot and between the different manufacturers. 3 IVIG lots of different manufacturers were excluded from administration to patients with blood group A since isoagglutinin titers of 64 were measured. For the used IVIG-preparations it can be assumed that their isoagglutinin content met the requirements of the European Pharmacopoeia. 9 out of 12 patients received the same preparation from one manufacturer during the whole treatment. In three patients, IVIG preparation was changed to another manufacturer due to repeated nausea, vomiting and chills during the first minutes of IVIG application. Hemoglobin levels that were determined on the occasion of each treatment day in patients with blood group O and blood group non-O are shown in Fig. 2. In pregnant women with blood group non-O, hemoglobin levels dropped after the first two treatment episodes to a significantly lower level compared to patients with blood group O (*p *= 0.0013). Nevertheless, the hemoglobin values in non-O patients remained stable on a lower level throughout further treatment (Fig. 2). Analysis of the hemoglobin levels showed no severe hemolysis (> 2 g/dl) between consecutive treatments. Side effects are summarized in Fig. 3. Headache was the dominant adverse event (92% of cases), especially after the first IVIG administrations, despite the patients were advised to drink sufficiently (1.5–2.0 l) before and during IVIG infusion. Headache was followed by nausea and vomiting (42%), exanthema (25%) and chills (25%), hypertension (17%), and hypotension (8%). Delivery was at 38 weeks of gestation (median, range 31–40), 25% of woman had a vaginal delivery, and 75% gave birth by caesarean section due to obstetrical reasons (Table 1).

**Table 1:** Overview of included patients, antibody specificities, start and number of treatments, mode of delivery and characteristics of the newborns like APGAR index, birth weight and platelet count

Baseline characteristics	
Patients with FNAIT history and treatment (*n*)	13
Included patients non-invasive treatment (*n*)	12
Age of the patient (years, mean)	34 [range 26–40, 95% CI]
Incompatibility	
Anti-HPA-1a	(*n*) = 11
Anti-HPA-15a	(*n*) = 01
Start of treatment (median)	20 weeks [range 20–31, 95% CI]
Number of treatment (median)	15 [range 6–19, 95% CI]
Delivery (median)	38 wks [range 31–40, 95% CI]
Mode of delivery	
Vaginal	(*n*) = 3
Primary caesarean	(*n*) = 5
Secondary caesarean	(*n*) = 4
Emergency caesarean	(*n*) = 0
Birth weight (median)	3010 g [range 2635–3400, 95% CI]
APGAR score	
8 or > 8 at 5 min	(*n*) = 12
< 8 at 5 min	(*n*) = 0
Sex of the child	
Male	(*n*) = 6
Female	(*n*) = 6
Blood platelets, nadir (median)	
Prophylaxis	187 × 10^9^/l [range 22–239, 95% CI]
Index case	17 × 10^9^/l [range 10–48, 95% CI]
ICH	
Index case	(*n*) = 1
With IVIG-therapy	(*n*) = 0

### Outcome of fetus/newborn

The median birth weight was 3010 g (median, range 2635–3400 g, 95%CI). The gender ratio was balanced. None of the newborns had APGAR scores below 8 at 5 min. The median platelet count at birth was 187 G/L (range 22–239 G/L, 95% CI) (Table 1). In three cases, the platelet count was lower than 50 G/L and a single antigen negative platelet transfusion was given in two of these cases. Two of the treated newborns had platelet counts clearly below 50 G/L and one of these newborn was delivered in 31th week of gestation and ICH in index pregnancy occurred.

**Fig. 4:**
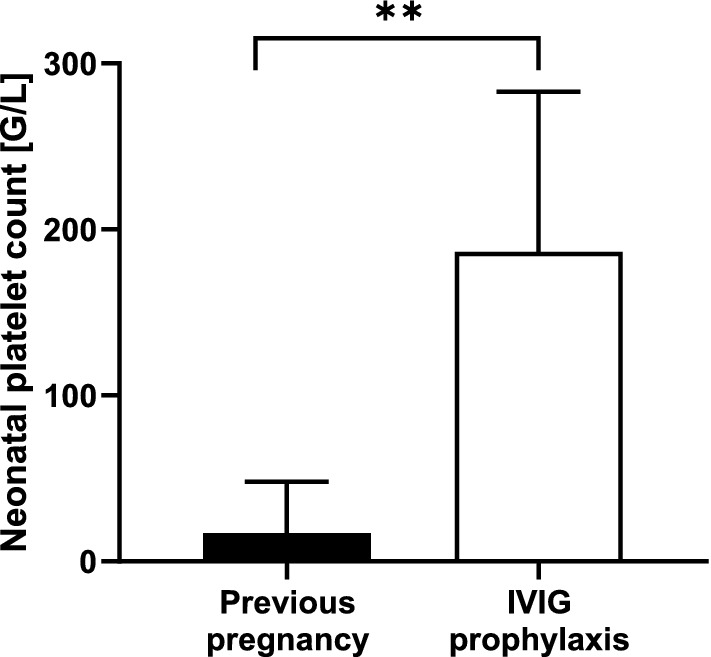
Distribution of neonatal platelet counts from the previous pregnancy and neonatal platelet counts from the pregnancy managed with maternally administered intravenous immunoglobulin (IVIG). Median, 95% CI, **p value 0.0058, *p* value is based on Mann–Whitney test

Due to a clinical decision the third newborn was not transfused (platelet count of 40 G/L, no bleeding signs, gestational age of 38 weeks, no ICH in index pregnancy). Platelet count was in all cases higher compared to index pregnancy (187 G/L vs 17 G/L, median, *p* = 0.0058) as shown in Fig. 4. In 1 neonate mild bleeding signs (petechiae) occurred (platelet count 235 G/L). Cranial ultrasound was performed in all neonates. No ICH was diagnosed in newborns after IVIG prophylaxis, compared to one ICH in preceding pregnancies (Table 1).

## Discussion

A completely non-invasive management of pregnant women with a history of FNAIT by non-invasive prenatal diagnosis of fetal HPA status, and fetal bleeding prophylaxis by weekly IVIG administration is safe and effective. Our retrospective case series showed no severe maternal or fetal complication: no emergency caesarean section was necessary and no ICH or severe bleeding occurred. All neonates showed higher platelet counts compared to the index case. This is in contrast to the results of a case series [10] which reported that weekly antenatal maternal IVIG infusions did not change the fetal/neonatal platelet count. These authors concluded that IVIG prophylaxis was not effective at all. However, this protocol included repeated diagnostic fetal blood sampling (median 7 per fetus). Fetal blood sampling is known to booster maternal antibody response by feto-maternal transfer of blood cells and we speculate that this booster effect may have antagonized the effect of IVIG.

The first non-invasive treatment of pregnant women with a history of FNAIT using maternal IVIG infusion was reported in 1988 [9]. A change to a completely non-invasive antenatal management was proposed in 2007 by the group of Oepkes et al. [11]. The data of this case series showed that non-invasive management is effective. A recent systematic review concluded that first-line antenatal management in FNAIT is weekly maternal IVIG administration [5]. Our case series demonstrated that a non-invasive strategy was effective in all cases, i.e. the cord blood platelet count of all neonates was higher compared to the platelet count of the affected sibling of the previous pregnancy. Relevant fetal or neonatal bleedings did not occur. The assumption that the severity of FNAIT increases with subsequent pregnancies for example shown by the data of Kamphuis et al. [12] was not observed and has recently been doubted by Tiller et al. who examined the natural course of FNAIT in subsequent pregnancies and showed that in two-thirds of cases, the younger siblings had unchanged or higher platelet counts at the time of delivery [3].

### Non-invasive fetal diagnosis of HPA genotype

A completely non-invasive strategy requires to determine whether the fetus is at risk and carrier of the platelet antigen against which maternal anti-HPA antibodies are directed. In this case, non-invasive prenatal genotyping is needed. Analysis of cffDNA may provide earlier diagnosis of fetal genotype than current invasive techniques. As cffDNA is found in maternal blood, sampling carries no associated risk. We recommend non-invasive fetal diagnosis of the HPA genotype by next-generation sequencing of cffDNA [7].This allows for quantification of the fetal DNA fraction. In case of a negative test result and low fetal fraction (< 4%) it may be necessary to repeat non-invasive testing in a higher week of gestation since fetal DNA fraction increases throughout pregnancy.

### Invasive diagnostic and prophylactic strategies

Invasive antenatal treatments with ultrasound-guided (serial) fetal blood sampling (FBS) and intrauterine platelet transfusion are associated with severe adverse events like booster of alloimmunization, fetal bradycardia, bleeding complication in the fetus, intrauterine death or emergency Caesarean section [6]. Furthermore, platelet transfusions are needed regularly, due to the short life time of transfused platelets, increasing the risk for fetal loss. The most common complications of invasive diagnostics are hemorrhage, or bleeding, of the puncture site and cord hematoma. The risk of hemorrhage is greater if the fetus has low platelet counts. Other possible complications are bradycardia, abortion, leakage, infections or booster of the maternal alloantibodies. Fetal loss may also occur, especially in the presence of several risk factors [13].

### Adverse effects of IVIG prophylaxis

Administration of IVIG caused adverse events in pregnant women that were treated for FNAIT: severe hemolysis dependent on isoagglutinin content of IVIG preparation can occur [14, 15]. Herrmann et al. reported IVIG-related pancytopenia [16]. Blood group A mothers are more likely to develop anemia during IVIG treatment of FNAIT [17]. To further diminish the hemolytic risk of IVIG application, isoagglutinin levels of each lot were assessed before infusion of patients of blood group A, B, or AB. Immunoglobulin lots with anti-A or anti-B titer < 64 were accepted. Furthermore, serial total blood counts on the occasion of each consultation were performed. A significant drop of hemoglobin value was noted in pregnant women of non-O blood groups compared to patients with blood group O. However, the mild hemolysis was compensated in all patients and remained stable throughout pregnancy.

We recommend serial laboratory monitoring for hemolytic anemia and pancytopenia to avoid serious maternal complications. Other adverse effects of IVIG application were mild and well treatable. The commonest adverse effect of IVIG infusion was headache that occurred several hours after the first treatment sessions.

For FNAIT prophylaxis exists no approval of an IVIG preparation [18]. Consequently, patients receiving IVIG should be carefully monitored at initial exposure. Rossi et al. [19] published effects on maternal lifestyle of FNAIT prophylaxis consisting of IVIG by one manufacturer and additional corticosteroid therapy. Three-quarters of 32 respondents from 64 treated patients reported that the treatments negatively affected their lifestyle. Thirty-one percent of women would not plan another pregnancy due to their experience and 22% were uncertain. Whether the commonly used dose of 1 g/kg/KG per week is needed or whether the dose could be reduced or should be increased remains unclear. Data from Paridaans et al. [20] and van der Lugt et al. [21] showed that a lower dose of IVIG (0.5 g/kg/bw per week) for patients with standard risk was not inferior to 1 g/kg per week. The lower dose could even more reduce side-effects and costs. However, more randomized controlled studies with risk stratification are needed.

The possible effects of IVIG for the fetal immune system are not fully investigated. In the follow-up study of Radder et al. [22] no clinically apparent adverse effects were found in 36 children aged > 5 years but larger prospective studies are needed.

### Delivery management

Our retrospective case series showed no severe maternal complication: no emergency Caesarean section was necessary. For the management of birth controversies exist. A conservative protocol was reported by van den Akker et al. [23]: vaginal delivery in FNAIT pregnancies without ICH in a previous child was safe. No diagnostic FBSs were carried out. However, induction of labor at 38th week of gestation was performed and assisted vaginal delivery was considered contraindicated. Caesarean section was only performed for obstetric reasons. It is generally agreed that in high-risk pregnancies (a sibling with ICH in a previous pregnancy), Caesarean section should be recommended [24]. However, a recent recommendation by Liebermann et al. [25] came to the conclusion that no trials evaluating the mode of delivery are existing. But forceps and vacuum-assisted delivery should be avoided.

## Conclusion

A non-invasive management of FNAIT consisting of prenatal diagnostics using cffDNA and IVIG prophylaxis in pregnancies of women with a history of FNAIT is effective to prevent fetal/neonatal bleeding complications. The adverse effects of weekly IVIG administration should be monitored and are common, but not severe.
